# Effects of Metoclopramide on Feeding Intolerance among Preterm Neonates; A Randomized Controlled Trial

**Published:** 2014-10-28

**Authors:** Mirhadi Mussavi, Khairollah Asadollahi, Ghobad Abangah

**Affiliations:** 1 Department of Pediatrics, Faculty of Medicine, Tabriz University of Medicine, Tabriz; 2 Department of Epidemiology; 3 The Research Center for Psychosocial Injuries; 4 Department of Gastroenterology, Faculty of Medicine, Ilam University of Medical Sciences, Ilam, Iran

**Keywords:** Meal Frequency, Vomiting, Prematures, Feeding Intolerance

## Abstract

***Objective:*** To evaluate the efficacy and safety of metoclopramide in the treatment of feeding intolerance in preterm neonates less than 36 weeks of gestational age.

***Methods:*** A randomized, controlled, masked cross-over study. A block of 4 randomizations was used. The “drug group” received intravenous metoclopramide before feeding and placebo group received placebo at the same time. The time to full enteral feeding and suspected adverse effects of metoclopramide, length of hospital stay or incidence of NEC or septicemia were the main outcome measures**.**

***Findings:*** Mean (standard deviation) of weight and Apgar score among metoclopramide and placebo groups were 1638.3±321 gr, 8.9±1.4 and 1593.3±318.8 gr, 8.8±1.3 respectively. Times to full feeding were significantly shorter in the metoclopramide group than in the control group (12.9±5.6 vs 17.0±6.3; *P*<0.0001) and also the numbers of withheld feedings were significantly lower (*P*<0.0001). According to the regression analysis, lower weight and placebo group were significantly related to increasing of lavage frequency, number of vomits, start time of feeding, number of feeding cessations and decreased feeding completion time (*P*<0.0001).No adverse effects of this treatment modality were observed in the two groups.

***Conclusion:*** Intravenous metoclopramide may be considered as an attempt in facilitating and treatment of feeding intolerance in preterm neonates.

## Introduction

Although enteral feedings are typically initiated for preterm infants during the first postnatal week, but advancing feedings to full enteral feeding is frequently difficult, especially in very low birth weight infants due to feeding intolerance and this may last for about 2-3 weeks. It necessitates prolonged parenteral nutrition with its attendant complications. Feeding intolerance is often attributable to functional immaturity of gastrointestinal motility in the small intestine, and more immature antroduodenal motor patterns in preterm than in term infants^[^^1^^- ^^4^^]^.

 Cisapride (Prepulsid) and erythromycin have not been approved for the treatment of neonates' feeding intolerance. The majority of pediatric patients must use alternative medications for treatment of gastro esophageal reflux disease and feeding intolerance, but there is no alternative therapy^[^^5^^-^^7^^]^. A drug that is frequently prescribed is metoclopramide (Reglan). Like many medications used in the pediatric area, using metoclopramide is not approved by the Food and Drug Administration, USA in those <18 years of age^[^^8^^,^^9^^]^. Although pharmacokinetic and pharmacodynamic studies of metoclopramide have been performed in the pediatric population, particularly in neonates, the available information is limited^[^^10^^-^^13^^]^. 

 In a survey of 57 NICUs in England and Wales, 53% reported using dopamine antagonists such as metoclopramide to treat GERD in premature infants. Approximately 25% of extremely low birth weight infants were discharged from the hospital with anti reflux medications^[^^17^^-^^20^^]^. In the literature, we couldn't find any clinical trial for improvement of enteral feeding in preterm infants with metoclopramide. Consequently, we conducted a randomized, placebo controlled trial to determine whether or not metoclopramide would benefit preterm infants with feeding intolerance and to assess the clinically significant adverse effects of this treatment modality.

## Subjects and Methods

By a clinical trial, all preterm infants admitted to neonatal care unit of Mustafa Khomeini Hospital in Ilam city, Iran, between March 2012 and August 2013, were sequentially enrolled. Inclusion criteria comprised: gestational age 29 to 36 weeks at birth, birth weight 900 to 2,000 gr, postnatal age at least 3 days, not achieving full enteral feeding volumes (150 ml/kg/day) within 5 days of the initiation of feedings, clinically stable (defined as normal blood pressure and no recurrent severe episodes of hypoxemia or bradycardia), and a gastric residual >30% of the feed volume given over the previous 3 hours on at least 2 occasions during a 24-hour period. Exclusion criteria included receiving mechanical ventilation, having a history of congenital neurological defect, major congenital anomalies, anatomic gastrointestinal abnormalities, suspected or proven necrotizing enterocolitis (NEC) within 7 days before the onset of feeding intolerance, cyanotic heart disease, clinically suspected or proven sepsis, metabolic or electrolyte disturbances and therapy with any of the following medications at the onset of feeding intolerance: fentanyl, ibuprofen, or pancuronium. During 18 months of study period, 120 sequential neonates fulfilled including criteria and participated in the trial. Feeding intolerance was considered if an infant had vomiting, severe abdominal distention (>15% of the baseline abdominal girth), having gastric residuals >30% of fed volume, or having frank blood in the stools. Feeding volume was not increased for 24 hours if feeding intolerance was present. The study drug (metoclopramide or placebo) was discontinued if an infant experienced any of the following complications: lethargy, irritability, diarrhea, dystonia or seizures. The study design was approved by the Ethics Committee in Ilam University of Medical Sciences, with IRB number EC/92/H/158, and informed consent was obtained from the parents of all neonates. All infants entering the study remained in the trial until they had reached full enteral feeding. 

 A randomized, controlled, masked cross-over study was performed. Each neonate was randomly assigned to one of the 2 study groups of metoclopramide or placebo by staff who was not involved in the infant's care. A block of 4 randomizations was used to ensure a balance of infants in each allocation. The allocation concealment was kept in an opaque sealed envelope, and the investigators, the patient care team, and the assessors were blinded to the treatment allocation. All of the subjects followed the same feeding protocol.

 The “drug group” received intravenous metoclo-pramide 30 minutes before feeding and placebo group received placebo (sterile water for injection) at the same time. Study medications were administered intravenously. Metoclopramide was given as a dose of 0.13 mg/kg/dose every 8 hours. The first dose of metoclopramide was given with the first feeding after enrollment. Intravenous preparations were used because they were clear, simpler and more practical than the oral route. Placebos of the same volume and color were administered for those in the placebo group. Both drug and placebo were code-numbered and prepared by a staff member who was not involved in the neonate's care during the study period and no other prokinetic agents were allowed.

 Parenteral nutrition was started for neonates at 3 days of life with amino acid (0.5 g/kg) and lipid (0.5 g/kg). The doses of both nutrients were subsequently increased in increments of 0.5 mg/kg/day up to a maximum of 3 g/kg/day. The initial glucose infusion rate was 4 to 8 mg/kg/min to a maximum of 10 to 12 mg/kg/min to maintain blood sugar concentrations within the normal range. Enteral feeding was initiated at the day 3 or 4 of life when the infants were clinically stable. Infants were fed by their own mothers’ milk. Enteral feeding was given through an orogastric tube as an intermittent bolus in 30 to 60 minutes, beginning with 10 to 20 ml/kg/day and increasing in increments of 10 to 15 ml/kg/ day for infants <32 weeks of gestation and 15 to 20 ml/kg/day for infants >32 weeks. 

 All infants were examined at least twice a day and closely monitored for drug side effects, vomiting, diarrhea, abdominal distention and volume of gastric residual. Gastric aspirate was measured every 3 hours before each feeding. Abdominal circumference was measured before feeding at 12-hour intervals and an increase in abdominal circumference of 1.5 cm over a 12-hour interval was considered abnormal. Any vomiting or gastric residual was recorded. The enteral feeding was stopped if vomiting occurred more than twice in 24 hours or there were clinical signs and symptoms suggesting NEC or any intra-abdominal pathology. An isolated incidence of bile-stained or blood-stained gastric aspiration with normal physical examination was not an indication for stopping feeding. The duration of withholding feeding was at the discretion of the attending physician. Enteral feeding and metoclopramide administration was resumed as soon as the aforementioned signs and symptoms subsided. The resumed feed was started at half the volume given before the feeding was withheld. Clinicians were asked not to make changes in feeding regimens (continuous vs bolus, NGT vs OGT) or type of feeding (breast milk versus formula milk) during the study period. Increasing the volume of feedings and number of feedings were the same in both groups.

 The primary outcome was the time to full enteral feeding (150 ml/kg/day). Secondary outcomes were incidence of NEC or septicemia, length of hospital stay, suspected adverse effects of metoclopramide. Data were expressed as means, frequencies, ranges, and percentages. Student t-test was used to compare the mean of different variables in treatment and placebo groups and X^2 ^or Fisher exact test in case of categorical data appropriately. ROC curve was applied to detect the most relevant variable affected by test variables. SPSS software version 11.0 (SPSS Inc, Chicago, Ill) was used for all statistical tests. Using a prevalence of 8% and 95% CI the total sample size needed for this study was 113 neonates; however, for better comparison of the variables, 120 neonates were finally estimated. *P* value less than 0.05 was considered as statistically significant.

 A consort checklist regarding this trial is submitted as attachment to this article.

## Findings

The study was conducted from March to August 2013. There were 120 neonates who were eligible for the study. The baseline characteristics of study infants and investigated variables are summarized in Table 1. 

**Table1:** Characteristics and different investigated variables of neonates that participated in metoclopramide study

**Variable **	**Metoclopramide**			**Placebo**			***P. *** **value**
	**Number**	**Mean (SD)**	**Range**	**Number**	**Mean (SD)**	**Range**	
**Number (boys/girls)**	60 (51/9)	-	-	60 (29/31)	-	-	0.0001
**Weight (gr)**	-	1638 (321)	1100-2000	-	1593 (318)	1000-2000	0.4
**Apgar score**	-	8.85 (1.36)	6-10	-	8.78 (1.3)	6-10	0.7
**Lavage **	n=21	0.47 (0.72)	0-3	n=37	1.98 (2.2)	0-8	0.0001
**Vomiting**	n=33	0.7 (0.74)		n=45	2.1 (1.98)	0-8	0.0001
**Feeding cessations **	n=22	0.55 (0.83)	0-3	n=40	2.3 (2.6)	0-12	0.0001
**Feeding start time (hrs)**	-	4.5 (2.3)	2-11	-	6.2 ( 1.99)	2-9	0.0001
**Feeding completion time (hrs)**	-	12.95 (5.6)	6-25	-	17.02 (6.03)	5-28	0.0001

SD: standard deviation

The reasons clinicians planned to start metoclopramide medications for study participants were as follows: vomiting associated with feedings and the need for gastric lavage prior to feeding. There was a significant difference between mean feeding completion times of metoclopramide group compared to placebo group (*P*<0.0001). The efficacy of metoclopramide was also identified by a reduction in the mean frequency of vomiting, lavage, feeding cessation times and feeding start times among metoclopramide group compared to placebo group (*P*<0.0001, *P*<0.0001, *P*<0.0001 and *P*<0.0001, respectively). According to the Student t-test results there was no significant difference between mean weight and Apgar score of metoclopramide and placebo groups (*P*=0.4, *P*=0.7 respectively). According to the regression analysis, lower weight and placebo group were significantly related to increasing of lavage frequency, number of vomits, feeding start time, number of feeding cessations and decreased feeding completion time (*P*<0.0001). No significant differences were observed in episodes of sepsis, necrotizing enterocolitis, adverse effects of this treatment modality and cholestasis between the two groups. Discriminate analysis and the relevant ROC curve were applied for different variables in metoclopramide group, among which the shown variables in Table 2 appeared the most appropriate. 

**Fig. 1:**
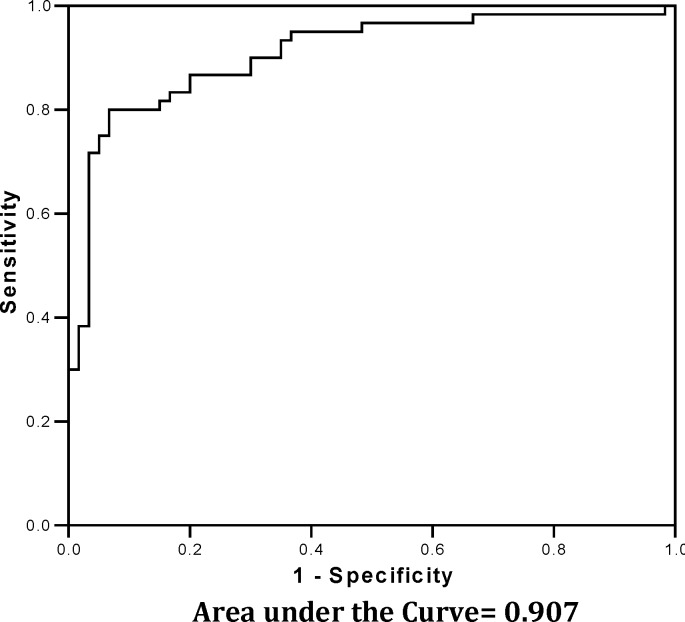
ROC curve for different variables applied in the metoclopramide group

**Fig. 2:**
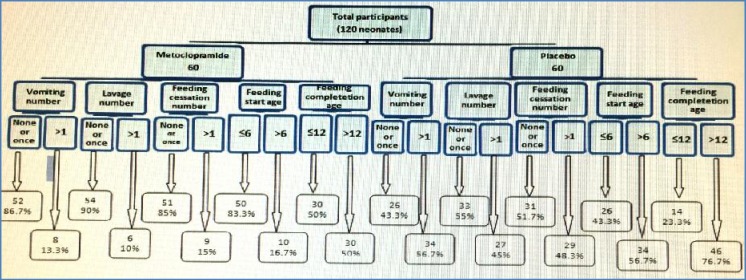
Flow algorithm of grouping and frequency rates of investigated variables among neonates participated in Ilam clinical trial

 The ROC curve applied for this variables group is shown in Fig. 1 with an excellent ROC curve and an AUC about 0.91. According to Fig. 1, ROC curve revealed a higher frequency of disorders for studied variables among neonates in the placebo group compared to those in the treatment group with a high AUC (0.907) significantly. That is, the ability of the test to correctly classify those with and without the different disorders, among both treatment and placebo groups. According to the results of this trial (Fig. 2) the frequency rate of disorders were notably reduced among metoclopramide group compared to the placebo group.

According to the results of this study, there was a significant difference between girls and boys for all studied variables and neonates with weight less than 1500 gr showed more vomiting and started or completed their feedings later than those weighing more than 1500 gr significantly (Table 3). Neonates with Apgar score more than 8 did not show any significant difference for studied variables except for age of feeding initiation that was shorter in those with Apgar score more than 8 significantly.

## Discussion

One of the major challenges of neonatologists in clinical practice is managing preterm infants to attain full enteral feeds without complications such as necrotizing enterocolitis, parenteral nutrition-related septicemia or cholestasis^[^^11^^]^.

**Table 2:** Weighting given to each variable in discriminate analysis

**Variables**	**Coefficient**
**Weight**	1.32
**Feeding cessation number**	0. 7
**Feeding start age**	0.56
**Feeding completion age**	0.32
**Lavage number**	0.31
**Gender**	0.28
**Vomiting number**	0.26

Preterm infants have great difficulty tolerating enteral feedings, which are usually presented by gastric residuals, vomiting and abdominal distention^[^^12^^]^. In addition to the previous mentioned problems our study revealed a higher incidence rate of feeding intolerance among those with lower gestational age. Metoclopramide, a derivative of orthoprocainamide, is primarily metabolized (at the first pass through the gut wall or liver) to metoclopramide N-4-sulfate, but as much as 25–40% of the compound may undergo renal clearance. Neonates may be at the risk of development of adverse effects associated with metoclopramide, including lethargy, irritability, diarrhea, dystonia and seizures.

**Table 3:** Comparison between occurrence rates of different studied variables among neonates in the treatment and placebo groups upon demographic variables

**Variable**		**Times vomiting**			**Times lavage**			**Times Feeding ** **cessation**			**Feeding start ** **age (hours)**			**Feeding completion ** **age (hours)**		
		**≤1**	**>1**	***P***	**≤1**	**>1**	***P***	**≤1**	**>1**	***P***	**≤6**	**>6**	***P***	**≤12**	**>12**	***P***
**Gender**																
**Metoclopramide** **n (%)**	**Boy**	23	6	0.001	24	5	<0.001	22	7	<0.001	22	7	0.02	13	16	0.008
	**Girl**	29	2		30	1		29	2		28	3		17	14	
**Placebo** **n (%)**	**Boy**	22	29		28	23		28	23		19	32		12	39	
	**Girl**	4	5		5	4		3	6		7	2		2	7	
**Total**	**Boy**	55	35		52	27		50	30		41	39		25	55	
	**Girl**	33	7		35	5		32	8		35	5		19	21	
**Apgar score**																
**Metoclopramide** **n (%)**	**≤8**	16	7	0.7	19	4	0.9	16	7	0.6	15	8	0.04	4	19	0.5
	**>8**	36	1		35	2		35	2		35	2		26	11	
**Placebo** **n (%)**	**≤8**	7	20		12	15		8	19		14	13		3	24	
	**>8**	19	14		21	12		23	10		12	21		11	22	
**Total **	**≤8**	23	27		31	19		24	26		29	21		7	43	
	**>8**	55	15		56	14		58	12		47	23		37	33	
**Weight**																
**Metoclopramide** **n (%)**	**≤1.5 kg**	17	8	0.02	19	6	0.09	16	9	0.1	15	10	0.04	0	25	0.04
	**<1.5kg**	35	0		35	0		35	0		35	0		30	5	
**Placebo** **n (%)**	**≤1.5 kg**	2	26		6	22		5	23		14	14		0	28	
	**<1.5kg**	24	8		27	5		26	6		12	20		14	18	
**Total**	**≤1.5 kg**	19	34		25	28		21	32		29	24		0	53	
	**<1.5kg**	59	8		62	5		61	6		47	20		44	23	

This drug acts both centrally on the chemoreceptor trigger zone to reduce activity and peripherally on the stomach and small intestine^[^^4^^,^^14^^-^^17^^]^. Metoclopramide has been used partially in the neonatal period that measured gastric emptying in neonates. The results of these studies indicated that metoclopramide did not promote gastric emptying in the neonatal period and concluded that any reduction in vomiting that might accrue from the use of metoclopramide in the newborn was attributable to a central action^[^^12^^,^^21^^]^. 

 Cisapride, a widely prescribed prokinetic agent, but the evidence for the benefit of Cisapride in premature infants remains poor and is based largely on results from older children and adults. Recent studies in preterm and older infants have challenged the effectiveness of cisapride. Furthermore, greater awareness of the potential side effects of Cisapride, such as prolongation of the QT interval, necessitates a more careful approach to its use in preterm infants^[^^16^^-^^19^^,^^22^^]^.

 A study conducted the effects of metoclopramide on promoting enteral feeding in preterm infants with feeding intolerance. The results of this study indicated that feeding tolerance improved steadily after metoclopramide was initiated, and by 29 days the infants tolerated full enteral feeding. No child receiving metoclopramide developed any extrapyramidal neurologic symptoms, worsening of hepatic function, or necrotizing enterocolitis. They concluded that metoclopramide may have a role in the treatment of premature infants with enteral feeding intolerance^[^^23^^]^. The results of our study showed a significant decrease in feeding completion times and a reduction in the mean frequency of vomiting and gastric lavage among metoclopramide group. When the studied variables were evaluated by discriminate analysis and ROC curve the frequency of disorders classified into treatment group were significantly lower than in the placebo group and the frequency rate of studied variables and relevant differences between metoclopramide and placebo groups have been also shown in the flow algorithm in the results section. All these evaluations revealed a considerable effectiveness of metoclopramide application among low birth weight and preterm neonates with feeding problem at the early stages of life. 

 A study by Pons and others reported an improvement in the volume of gastric aspirates, daily weight gain, intestinal transit time, and volume of feeding in premature infants who were given metoclopramide 0.1 mg/kg/day intravenously. Metoclopramide increases the amplitude of peristaltic contractions in the esophagus, gastric antrum, and small intestine, elevates the resting tone of the lower oesophageal sphincter and stimulates gastric emptying^[^^24^^]^. The results of our study showed that intravenous metoclopramide has a significant effect in facilitating and treatment of feeding intolerance, reduction in the mean frequency of vomiting, lavage, feeding start times and enteral feeding completion time among metoclopramide group compared to placebo group.

 One of the limitations of the present study was lack of data related to serum levels of the drug and/or gastric emptying rates for comparison between serum levels of metoclopramide and gastric emptying rates. A future study with higher sample size, to investigate the relationship between serum level of metoclopramide and studied variables, with assistance of a clinical pharmacologist, can increase the quality and preciseness of detected results in the current study.

## Conclusion

According to the results of our study, the curative effects of metoclopramide could be considered as an attempt in facilitating and treatment of feeding intolerance in preterm neonates. This study was insufficient to either support or oppose the use of metoclopramide in infants; large blinded randomized clinical trials are needed to determine the efficacy or toxicity of metoclopramide in this population.
